# Externally Controlled Triggered-Release of Drug from PLGA Micro and Nanoparticles

**DOI:** 10.1371/journal.pone.0114271

**Published:** 2014-12-05

**Authors:** Xin Hua, Shengnan Tan, H. M. H. N. Bandara, Yujie Fu, Siguo Liu, Hugh D. C. Smyth

**Affiliations:** 1 State Key Laboratory of Veterinary Biotechnology, Harbin Veterinary Research Institute of Chinese Academy of Agricultural Science, Harbin 150001, China; 2 State Engineering Laboratory of Bio-Resource Eco-Utilization, Northeast Forestry University, Harbin, PR China; 3 College of Pharmacy, The University of Texas at Austin, 1 University Station, A1920, Austin, TX 78712, United States of America; King Abdullah University of Science and Technology, Saudi Arabia

## Abstract

Biofilm infections are extremely hard to eradicate and controlled, triggered and controlled drug release properties may prolong drug release time. In this study, the ability to externally control drug release from micro and nanoparticles was investigated. We prepared micro/nanoparticles containing ciprofloxacin (CIP) and magnetic nanoparticles encapsulated in poly (lactic-co-glycolic acid) PLGA. Both micro/nanoparticles were observed to have narrow size distributions. We investigated and compared their passive and externally triggered drug release properties based on their different encapsulation structures for the nano and micro systems. In passive release studies, CIP demonstrated a fast rate of release in first 2 days which then slowed and sustained release for approximately 4 weeks. Significantly, magnetic nanoparticles containing systems all showed ability to have triggered drug release when exposed to an external oscillating magnetic field (OMF). An experiment where the OMF was turned on and off also confirmed the ability to control the drug release in a pulsatile manner. The magnetically triggered release resulted in a 2-fold drug release increase compared with normal passive release. To confirm drug integrity following release, the antibacterial activity of released drug was evaluated in *Pseudomonas* aeruginosa biofilms in vitro. CIP maintained its antimicrobial activity after encapsulation and triggered release.

## Introduction

*Pseudomonas aeruginosa* (*P.* aeruginosa) is a Gram-negative bacterium belonging to the bacterial family pseudomonadaceae. *P.* aeruginosa is responsible for many types of infections which are difficult to treat, such as ulcerative keratitis, skin and soft tissue infections, pneumonias and urinary tract infections [1]. According to the report of United States Cystic Fibrosis (CF) Foundation Patients Registry (2004), 57.3% of all reported respiratory cultures in CF patients contained *P.* aeruginosa [2]. Ciprofloxacin hydrochloride (CIP) is a widely used antibiotic drug against a broad range of clinically relevant Gram-negative and Gram-positive pathogens infections. In initial stage of administration, oral CIP appeared to be effective. However, infections are often persistent and difficult to clear [3]. For example, urinary tract infections required twice daily at doses of 250 mg or once-daily extended-release ciprofloxacin; a current treatment regimen of cystic fibrosis requires ciprofloxacin at least twice-a-day administration at a dose of 300 mg for alternating 28-day on-off cycles [4]. It was reported that a sustained released antibiotic formulation may reduce inconvenience of patient and increase therapeutic efficacy [5], [6]. In addition, aminoglycoside therapy of cystic fibrosis lung infections may be realized if delivery of sufficient drug at sustained levels in and around the target infections could be achieved [7].

Poly (lactic-co-glycolic acid)-PLGA is one of the most successfully used biodegradable polymers for controlled drug delivery systems [8]–[13]. As a result, there is a significant amount of reported research on PLGA micro/nanoparticles for long term release using (water-in-oil-in-water) W/O/W methods [14], [15]. Both PLGA micro and nanoparticles have potential advantages depending on drug properties, disease characteristics, and desired administration requirements [12], [16]. If disease treatment focuses on delivery to the deep lung as an inhalation formulation, inhaled particles should have aerodynamic diameters between 1µm and 5µm [17].

Magnetic nanoparticles (MNPs) have drawn attention for their intrinsic magnetic properties and have therefore been applied as targeted drug delivery systems [18]–[20] or as hyperthermia agents in the field of cancer treatment [21]–[22]. When placed in an external applied magnetic field, MNPs show superparamagnetism [23]. This enables MNPs to potentially be targeted to a desired location of human body [24]. Furthermore, when placed in an external magnetic field, MNP can respond by causing local hyperthermic conditions [25] and/or result in mechanical disruption of polymer materials to cause drug release [26]. Previously we reported ability of magnetic nanoparticles to cause disruption of a biopolymer [26]. In addition, we also demonstrated that these effects could be achieved at much lower magnetic fields than previously reported with little to no hyperthermia or heating of the surrounding tissues under OMF [26]. Several researchers have focused on magnetically triggered release. Pradhan et al. [27] described thermosensitive magnetic liposomes for use in magnetic hyperthermia-triggered drug release to treat by thermo-chemo-therapy in cancers. A recent study reported by Hu et al. [28] demonstrated that a Yolk/shell capsules containing a volume/hydrophobicity transformable core and an ultra-thin silica shell have been prepared which can exhibited a triggering size shrinkage causing solid shells destruction and drug burst release when an external magnetic field applied. Moreover, incorporation of superparamagnetic particles into PLGA particles provides more opportunities for targeting efficacy and triggered release property. Recently, paclitaxel (PTX) and magnetic nanoparticles (MNPs) coencapsulated, PLGA-L-lysine-D-galactose (PTX-MNP-PLGA-Lys-Gal) nanoparticles preparation has been reported [28], and burst release of PTX can be triggered under near infrared (NIR) irradiation. Surprisingly, there is relatively few research papers published on the externally triggered drug release from PLGA particles [29]–[31]. Moreover, there is no references comparing triggered drug release characteristics between PLGA micro and nanoparticles under low energy magnetic fields. In this study, CIP encapasulated PLGA magnetic micro or nanoparticles have been prepared and their release properties under different condition have been investigated.

By combining the sustained release and biocompatibility advantages of PLGA micro or nanoparticles with the attractive properties of magnetic nanoparticles, a controlled and triggered drug delivery system could be developed. In these studies, CIP and MNP loaded into PLGA micro/nanoparticles allowed the investigation of sustained and triggered release properties of this system.

## Materials and Methods

### Materials

Poly (lactic-co-glycolic acid, 50∶50, Mw∼85,000) was purchased from Birmingham Polymer Inc. (USA). Ciprofloxacin·HCl was purchased from Letco Medical (USA). The magnetic nanoparticles used in these studies were nano-screenMAG-CMXR (Chemicell, Berlin, Germany), an aqueous dispersion of magnetic-fluorescent nanoparticles with a hydrodynamic diameter of 200 nm. The particles consist of a magnetite core which is first covered by a lipophilic fluorescence dye. A second layer envelops the particle with a hydrophilic polymer (carboxymethyl-dextran) which protects the particles against aggregation. In the manuscript we abbreviate these nano-screenMAG-CMX^R^ particles as CMX. All other chemicals were used as HPLC grade or extra pure grade.

### Preparation of CIP encapsulated in PLGA magnetic particles

CIP encapsulated in PLGA magnetic micro or nanoparticles were prepared by water-in-oil-in-water double emulsion evaporation method, sonicator 4000 (Misonix, USA) and polytron PT-MR 2100 benchtop homogenizer (Kinematica AG, CH) were used. Briefly, 310µL CIP (20 mg/ml) and 90µL CMX (25 mg/ml) was added dropwise into 4 ml Dichloromethane (DCM) containing 40 mg PLGA and sonicated for 2 min at 40% amplitude (24 cycles of 5 s) to get the primary water in oil emulsion. Next, to prepare CIP encapsulated in PLGA magnetic nanoparticles, 20 ml 2% Polyvinyl Alcohol (PVA) was added and sonicated in an ice bath for 2 min at 40% amplitude (24 cycles of 5 s) for stabilization of the emulsion. For CIP encapsulated in PLGA magnetic microparticles preparation, the secondary emulsification was performed by homogenizing the water-in-oil (W/O) emulsion with 20.0 mL 2% PVA solution at 15,000 rpm 10 min. All the samples were placed in orbital shaker over night to evaporate the organic solvent at room temperature. Washing the harvested samples with double distilled water was performed and then centrifuged at 4,000 rpm for 10 min. This washing procedure was repeated three times. For drug loading efficiency testing some samples were freeze-dried for 3 days.

### Particle size measurement

The size distribution of CIP encapsulated PLGA nanoparticles was determined by Dynamic light scattering (DLS) (Zetasizer Nano ZS, Malvern, UK). We used Sympatec Laser diffraction HELOS system equipmed with the cuvette attachment for CIP encapsulated PLGA microparticles size measurement (Sympatec, Germany).

### SEM observation

The surface and morphology of particles were evaluated by Zeiss Supra 40 VP Scanning Electron Microscope (SEM) (Carl Zeiss, Germany). Samples were prepared by placing a droplet of an aqueous suspension onto a carbon tape and dried over night.

### Drug content and loading efficiency

To determine the drug content, 10 mg particles were dissolved in 70% dimethylsulfoxide (DMSO) and sonicated for 2 h. CIP concentration was measured using UV-spectrophotometer (Tecan infinite M200, Switzerland) at 276 nm. Drug content and loading efficiency were as follows:
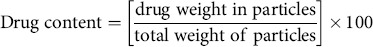


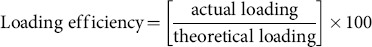



The theoretical loading means the point where all the supplied ciprofloxacin was encapsulated in the spheres.

### Drug release study

For normal drug release (i.e. without magnetic fields), 100 mg particles was suspended in 1 ml phosphate buffered saline (PBS, pH 7.4, 0.1 M).1 ml CIP encapsulated in PLGA micro or nanoparticles were placed in dialysis bag (MWCO:12,000–14,000) in 15 ml microtubes with an additional 9 ml PBS buffer, yielding a total volume of release medium of 10 ml. Release was performed in an incubated shaker at 37°C and 100 rpm. At regular intervals, to ensure sink conditions were maintained, 1 ml samples was taken and replaced with an equal volume of fresh PBS. Triggered released using an external magnetic field was also assessed with the same method The microtube was placed in a custom-built oscillating magnetic field apparatus (Magnetherm, NanoTherics Ltd, UK) for exposure 1 to 6 hours. The nominal frequency of the oscillating magnetic field was 355 kHz with an amplitude of 0.56 kA/m, and a DC power supply voltage was 26V. 500µl samples were taken and replaced with an equal volume of fresh PBS at predetermined time intervals. Samples were centrifuged to then further purified through magnetic MACS Separation 20µ columns to remove any remaining magnetic particles (Miltenyi Biotec GmbH, USA) and allowing collection of the released CIP. The concentration of released drug was measured with UV-spectrophotometer at 276 nm. All experiments were performed in triplicate. The cumulative CIP release was calculated as:
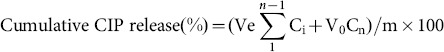



Where V_e_ is the amount of release media taken out every time (1 ml), V_0_ is the amount of release medium (10 ml), C_i_ is the concentration of CIP released from particles at intervals of i, m is the mass of drug used for release and n is the replacement times.

### Antibacterial activity of ciprofloxacin-encapsulated PLGA particles

*P. aeruginosa PAO1* was kindly gifted by Dr. Marvin Whitely, from the Department of Molecular Genetics and Microbiology at The University of Texas at Austin. *P. aeruginosa* biofilms were developed as described by Bandara [32]. In brief, *P. aeruginosa* suspension 100µl (10^7^organisms/ml) was dispersed into wells of 96-well plate at time 0 and incubated in an orbital shaker (75 rpm) at 37°C for 90 min for the adhesion phase. Then, wells were washed with sterile PBS twice, and 200 µl of media were added and cells were incubated for 24 h (37°C, 75 rpm) for the *P. aeruginosa* biofilm formation phase. At the end of incubation, the wells were again washed twice with sterile PBS. 198µl of media and 2µl of serially diluted test and control samples were added to the preformed mature *P. aeruginosa* biofilms and incubated for 24 h (37°C, 75 rpm). Following incubation, the supernatant was removed, the wells were washed twice with sterile PBS and a standard XTT reduction assay was performed as described by Jin *et al* [33] to measure the metabolic activity of biofilms. The color changes were measured with a microtiter plate reader (Tecan infinite M200, Switzerland) at 492 nm. All assays were carried out at least in triplicate on three different occasions.

### Statistics

Significant differences were calculated using a paired Student's t-test. Values of p<0.05 were considered significant.

## Results and Discussion

### Particle Characterization

**Figure 1:**
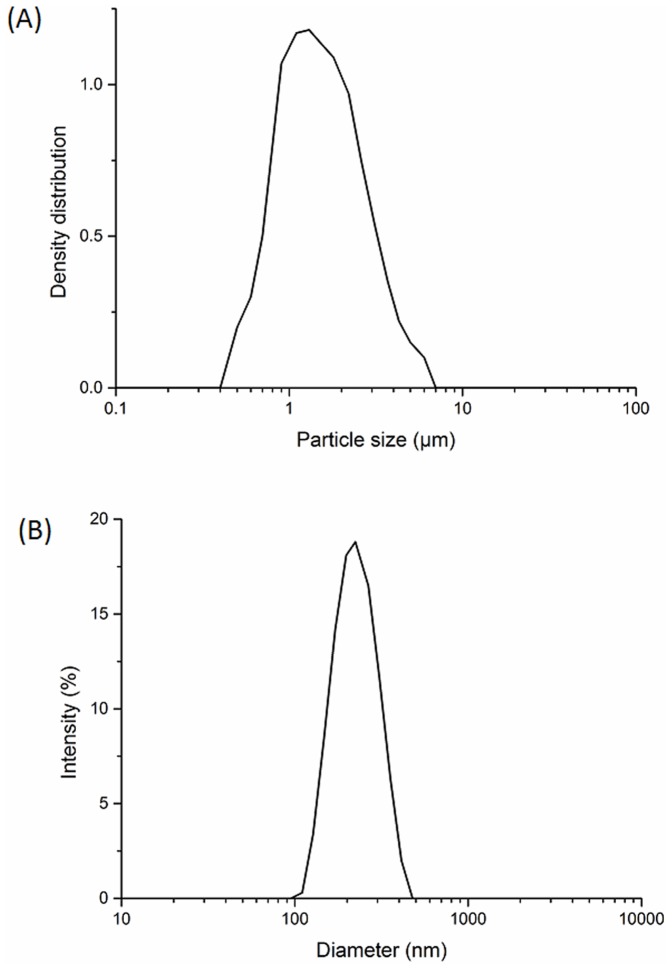
Particle size distribution of CIP encapsulated in PLGA magnetic microparticles (A) and nanoparticles (B).

**Figure 2:**
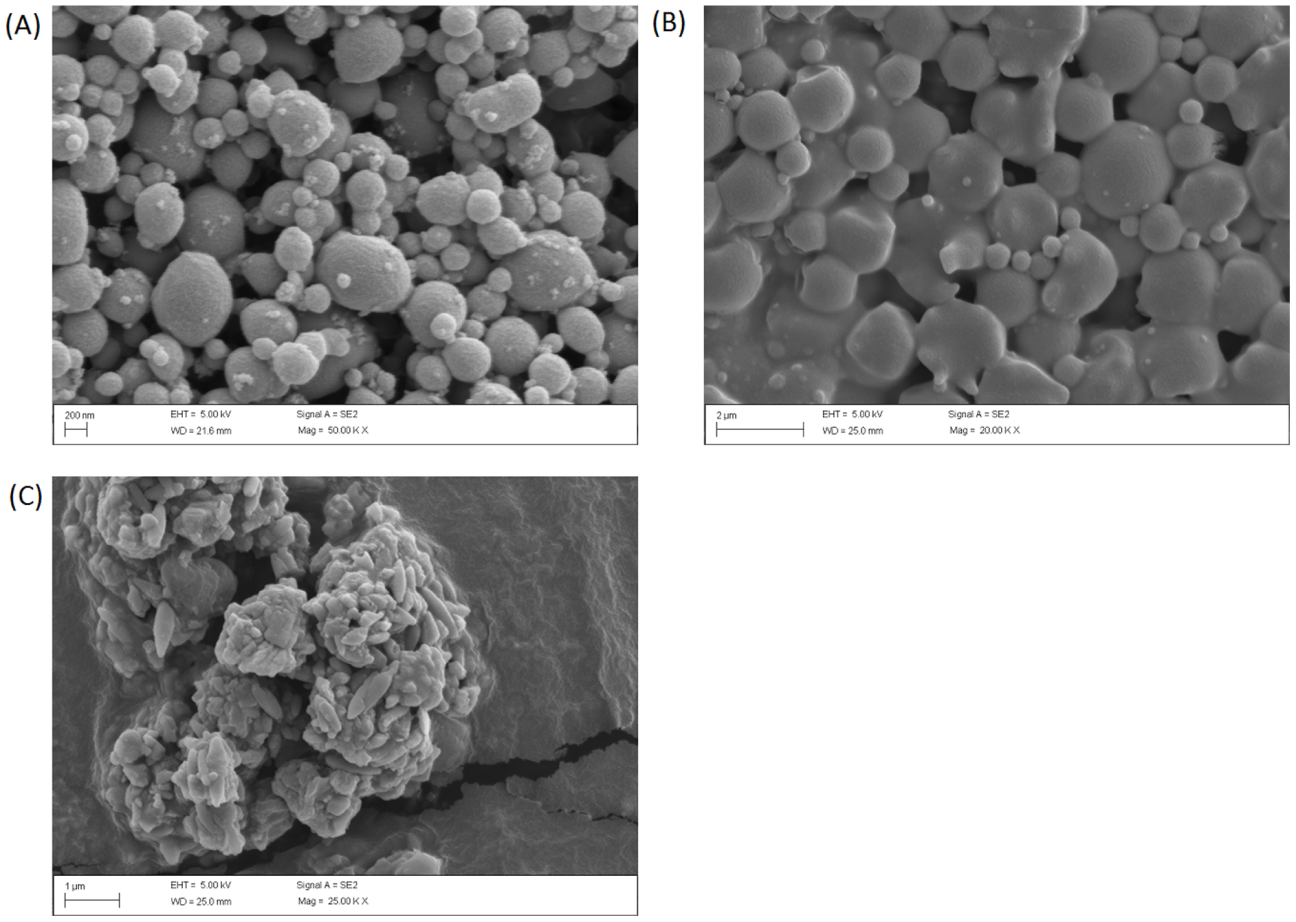
Surface morphology of CIP encapsulated PLGA magnetic particles by SEM. (A) CIP encapsulated in PLGA magnetic nanoparticles (B) CIP encapsulated in PLGA magnetic microparticles (C) CMX.

CIP encapsulated in PLGA magnetic particles were prepared using a double emulsion technique. Two different particles sizes were produced using either sonication or homogenization as shown in Table 1. Nanoparticles and microparticles obtained had average diameters of 221 nm (sonication) and 1.5µm (homogenization) respectively. Fig.1 shows the typical particle size distribution of CIP encapsulated in PLGA micro or nanoparticles. These results are consistent with the particle size analysis shown in Fig.2A and B. The size distributions were between 95–477 nm for the nanoparticles and 0.5–6µm for the microparticles respectively (Fig.2A and B). PDI of both nano- and microparticles were less than 0.5, indicating size distributions for both nano- and micro- particle were narrow.

**Table 1:** Characterization of CIP and CMX encapsulated PLGA particles.

	Drug Contents	Loading Efficiency	Particle Size
	(%, w/w)	(%, w/w)	(Mean ± SD)
nanoparticles	2.78	54.67	220.9+7.4 nm
microparticles	3.65	76.88	1.45±0.74µm

According to previously published data [34], 2% PVA was chosen as an emulsion stabilizer for preparation of PLGA particles. It can help improve the entrapment efficiency in O/W emulsion methods [35], [36]. Although PVA may result in toxicity in certain conditions, recent evidence suggest magnetic nano particles are less or no toxic when PVA coated [37], [38]. Both nano and microparticles were spherical in morphology with relatively smooth surfaces (Figure2 A, B), which was in accord with previous reports [35]. As shown in table 1, the loading efficiency was 76.9% for microparticles, while it was only 54.7% for nanoparticles. Compared with microparticles, the lower internal dimensions of nanoparticles were attributed as the main reason of lower loading efficiencies. CIP, a water soluble drug, may have increased drug loss during sonication and second emulsion steps according a literature report [39]. However, both of the loading efficiencies observed in our studies were>50%, and were considered successful.

### Passive Drug release from PLGA particles

**Figure 3:**
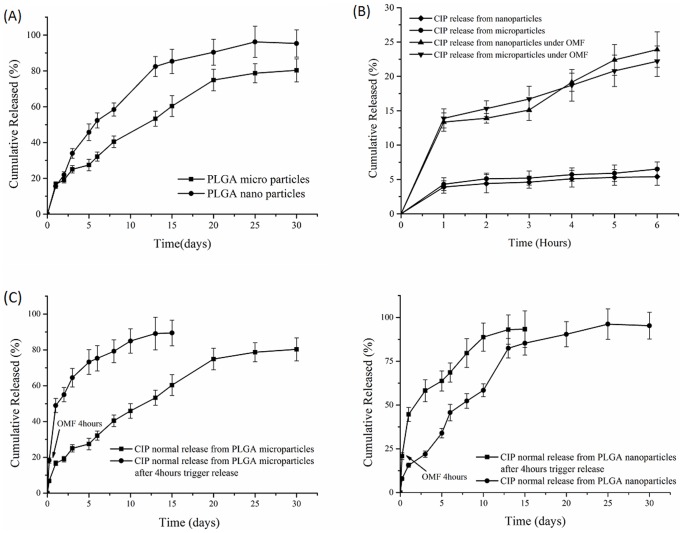
Drug release from PLGA magnetic micro/nanoparticles. (A) Drug release in PBS buffer from PLGA magnetic micro/nanoparticles at 37°C and shaking at 100rpm. (B) Drug release from PLGA magnetic micro/nanoparticles under OMF and control drug release from PLGA magnetic micro/nanoparticles at 20°C without OMF. (C) Drug release from PLGA magnetic microparticles under OMF for initial 4hours then released in PBS buffer at 37°C and shaking at 100 rpm for another 15 days. (D) Drug release from PLGA magnetic nanoparticles under OMF for initial 4 hours then released in PBS buffer at 37°C and shaking at 100 rpm for another 15days.

**Figure 4:**
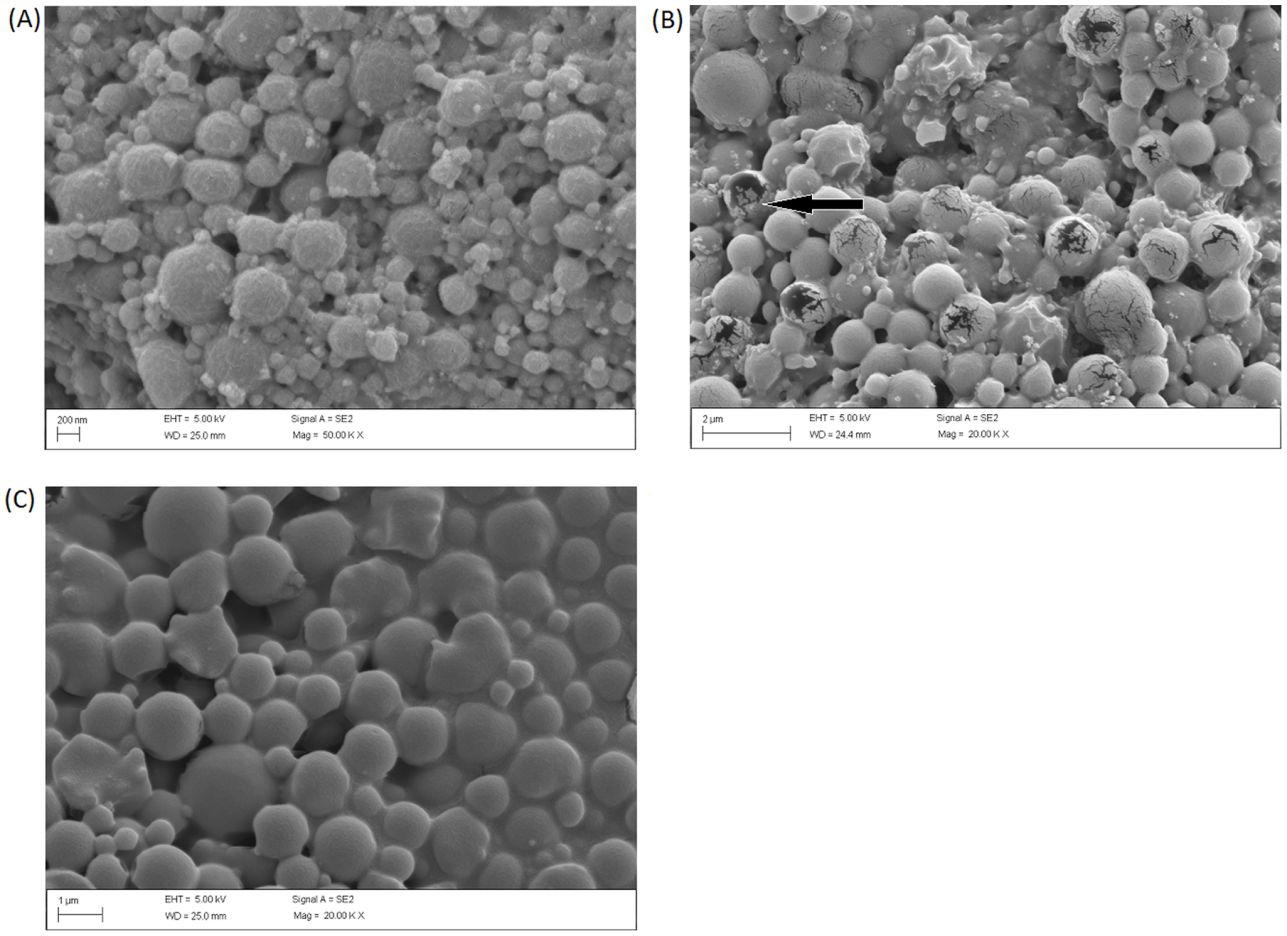
Surface morphology of CIP encapsulated PLGA magnetic particles after trigger release under OMF for 4hours by SEM. (A) CIP encapsulated in PLGA magnetic nanoparticles after trigger release under OMF. (B) CIP encapsulated in PLGA magnetic microparticles after trigger release under OMF (arrow indicating crack on the surface of microparticles). (C) CIP encapsulated in PLGA microparticles after trigger release under OMF.

CIP release profiles from the various particle systems produced were investigated. Under passive conditions, prolonged release for 30 days from all PLGA particles was observed. Surprisingly, as shown in Figure 3A, similar release kinetics were observed between nanoparticles and microparticles. They both showed initial burst release in the first 2 days, release rates (total release amount of drug per hour or day) of nanoparticles and microparticles were 304.4 µg/day and 340.8 µg/day respectively. Following this “burst release” the next 4 weeks exhibited sustained release from the particles. The two systems showed linear release rates from 2–14 days, with release rate of 146.8 µg/day for the nanoparticles and 125.6µg/day microparticles. The nanoparticles had slightly greater release rates from 2–14 days than the microparticles as expected, due to surface area differences. These different rates lead to significant differences in total drug release for the two systems, 63.4% versus 41.3% from 2–14 days. After 14 days, there appeared to be little difference in release rates between the two systems. For the microparticles, drug release was linear up to the 20^th^ day, during which the release rate was 106.6µg/day. However, over the next 10 days, drug release from the microparticles was observed to decrease to 18.9µg/day. For nanoparticles, after the 15^th^ day, drug release was 18.5µg/day. The possible reason of the initial burst release was unencapsulated weakly adhering drug on the microparticles and nanoparticles surfaces [40], [41]. Nanoparticles showed the higher total drug release, 95% after 30 days. We hypothesize that the differences in the surface areas of the nano and microparticles may explain this. The surface area of a single sphere is πd^2^ (d is diameter of sphere) and the volume of a sphere is πd^3^/6; thus, the specific surface area per unit volume (S_v_) for a single spherical particle is 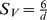; The specific surface area per unit mass (S_w_) for a single spherical particle is as below:
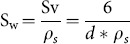



Here *ρ*_s_ is the density of sphere. We assumed the density of micro and nanoparticles was equal and calculated the ratio of theoretical specific surface of micro and nanoparticles. Using the formula above, the theoretical surface areas of the nanoparticle system was calculated to be 7 fold higher than the available surface area of the microparticles. Thus, the nanoparticles, with significantly higher surface areas would be expected to release drug more rapidly from the surface. However, it was suspected that the PLGA microparticles may have thinner walls than their nanoparticle counterparts. For example, as shown in the SEM micrographs, under certain treatment conditions, breakage of the walls occurred with the microparticles but not the nanoparticles (Fig. 4A and B). The thinner walls may explain why the nanoparticle release was not orders of magnitude different from the microparticle release as would be predicted by the specific surface areas.

As the encapsulation method for ciprofloxacin within the particles was via a W/O/W technique, we suggest that the drug release will mainly occur via drug diffusion out of particle surface, and partly due to PLGA hydrolysis mediated particle erosion/surface poration as described previously by Hirenkumar et al [42]. It can be confirmed by fitting data with Ritger-peppas model, which is often used to describe drug release from matrix systems of various geometries as the formula below:
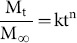



Where t is release time, M_t_/M_∞_ denotes the fraction of drug released at time t;K is the Ritger-Peppas release rate constant; n is indicated the mechanism of drug release. By fitting data using Ritger-Peppas model, the formulations of particles were as below:


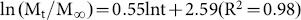
 for microparticles;


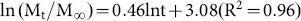
 for nanoparticles

Because the values of n were found between 0.45–0.89, thus both diffusion and polymeric chain relaxation were found responsible for drug release [43]. When n is close to 0.45 indicated that diffusion was the dominating mechanism of release. All the data was fitted using Origin8.0, which confidence interval of the default was over 95%.

In addition to the model drug, magnetic nanoparticles were also loaded into the PLGA particle systems. To confirm MNPs incorporation into the PLGA particles, both magnetic separation and size separation steps were performed on the formed particles. Following magnetic separation through the magnetic columns (as described in the methods section), a 0.22µm filter was used to separate unbound CMX from the CIP encapsulated in PLGA magnetic particles. Since the MNP we selected for these investigations were also fluorescent, they were quantified at excitation 578 nm, emission 613 nm and confirmed that magnetic nanoparticles has been incorporated into PLGA micro/nanoparticles.

### Magnetically Triggered Drug release from PLGA particles

As introduced above, MNPs can be externally manipulated in several different ways. In these experiments we wanted to test the hypothesis that MNPs co-loaded PLGA particles could be used to triggered drug release or increase release rates using an external low energy oscillating magnetic field. In these studies, drug release was performed in PBS buffer at 20°C as a control (OMF treatment group was water cooled in the magnetherm, temperature is 20°C). Separately, microparticles and nanoparticles suspended in dialysis bag within a 15 ml microtube with PBS buffer were exposed to oscillating magnetic fields maintained water cooled in the magnetherm. Both particle types showed higher percentage drug release under OMF than controls (Fig 3C). It was notable that drug release percentage of microparticles was higher than nanoparticles after 4 hours. And this phenomenon did not show in control group. Surface cracks were observed on microparticles following OMF treatment (Fig. 4B) but were not observed in control particles. It appears, therefore, that increased release of drug from the microparticles during OMF induced drug release was caused by the mechanical poration of the microparticles by the activated MNPs. Microparticles surface walls may also have been sufficiently thin to allow poration by mechanical and/or thermal forces generated by the MNPs. However, the surfaces of the nanoparticles exposed to the same OMF were observed to be unbroken and uniform under SEM analysis. The total released drug of microparticles and nanoparticles under OMF at 6 hours were 23.9% and 22.2% respectively.

Poration of the particles may influence the drug release rates after the removal of the magnetic field, and could have enabled the particles to rapidly empty the drug contents. Thus, to test if micro/nanoparticles retained their sustained release ability after OMF treatment, we conducted drug release experiments from the PLGA magnetic particles after 4 h OMF treatments. Samples of micro/nanoparticles after OMF treatment were placed into dialysis bags with PBS buffer as the method to monitor drug release. Both micro/nanoparticles demonstrated sustained drug release profiles for 15 days (Fig. 3C and D). Drug release percentage of microparticles was higher than nanoparticles from 1 to 7 days. The total release amount under OMF was 89.4%, and higher than passive release from microparticles as reported above (80.3%). In future applications, it could be foreseeable that the drug loaded PLGA system described here could be targeted to a certain location and then, once desirable, drug release at high rates could be initiated using the external OMF to ensure therapeutic effect at the site of action.

**Figure 5:**
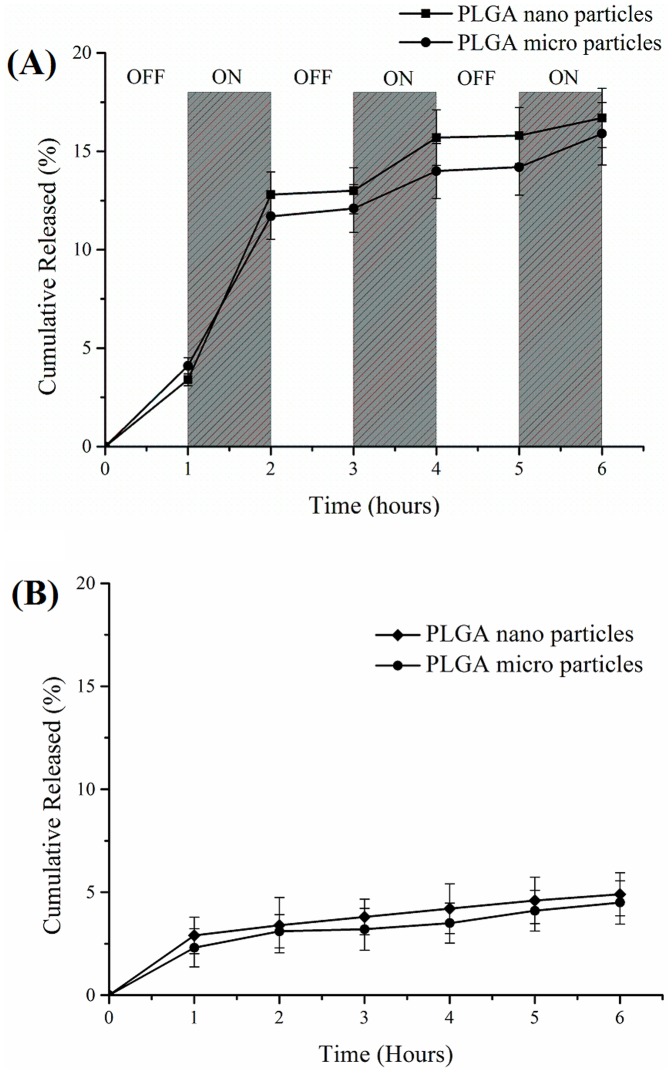
Drug release from PLGA magnetic micro/nanoparticles. (A) Switched turn-on and -off drug release under OMF. (B) Drug release with OMF off.

To further investigate this controllable release property, we conducted a “turn-on and -off switch” drug release experiment (Fig. 5). In these studies, an OMF was applied to the particles from 0–1 hours, turned off from 1–2 hours, switched on at 2–3 hours and so on, until 6 hours was reached (Fig. 5A). A control experiment of drug release was conducted in the same condition from 1 to 6 hour keeping OMF off as shown in figure 5B. It can be seen that during the “off” time periods, there was low or even no drug release observed. This phenomenon was also seen in control group, the total drug release amount was less than 5%. Drug release observed during the first “off” period was attributed to residual drug also observed in the control experiments. A significant increase in drug release during the “ON” time periods was observed, especially the first “on” period where 10% of drug was released, with the release rate 55.5µg/h for the microparticles and 52.3µg/h for the nanoparticles. While in the next two “ON” stage, average 4% of total drug was released less than the amount of first “ON” period. Because of partial drug weakly adhered to the surface of particles, drug was easily released under the effect of OMF and resulted in burst release in the first “ON” period. Drug release rates from micro and nanoparticles were lower during the second and third “off” periods (less than 4µg/h). Collectively, these studies showed the high degree of adaptability of using PLGA-magnetic particle systems as a controlled release platform for drugs like ciprofloxacin.

### Antibacterial activity of ciprofloxacin-encapsulated PLGA particles

**Figure 6:**
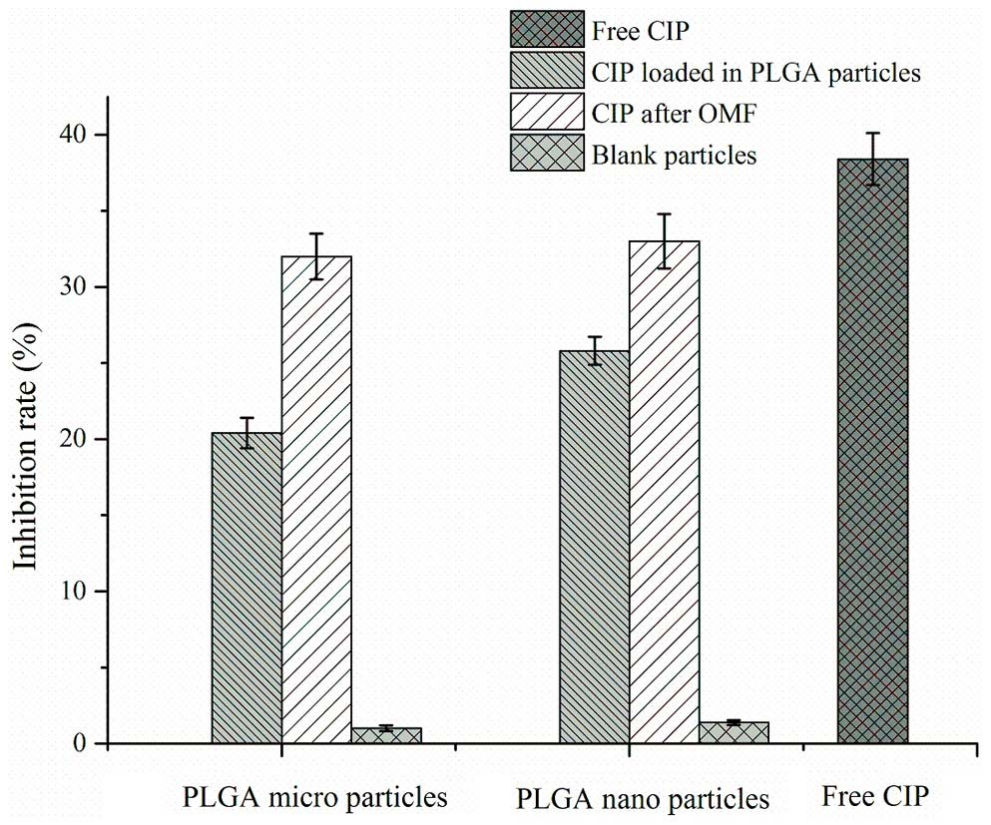
In vitro antibacterial activity against P. aeruginosa biofilms of free CIP and CIP encapsulated in PLGA magnetic micro/nanoparticles and of CIP release from magnetic PLGA micro/nanoparticles under OMF.

To ensure the functionality of the released drug during passive and triggered release and also assess the relative antimicrobial performance of the different particle systems developed, a biofilm treatment assay was conducted. *Pseudomonas aeruginosa* forms biofilms readily and is typical in chronic infections, and are persistent and hard to treat [44]. Thus, the inhibition of biofilm (An in-vitro biofilm assay in which the biofilm metabolic activity is assessed via XTT assay) is a useful indicator of the antibacterial activity of drugs/delivery systems. To test antibacterial activities of CIP-encapsulated PLGA particles, CIP-encapsulated particles were dosed to *P. aeruginosa* biofilms. Free CIP was a positive control while untreated *P. aeruginosa* biofilms served as a negative control. Bacterial growth was measured by UV-spectrophotometer and expressed as an OD values as according to established methods [33]. Figure 6 shows the antibacterial activities of free CIP and CIP-encapsulated particles. From Figure 6, empty micro/nanoparticles had insignificant *Pseudomonas aeruginosa* biofilm inhibition (less than 2%), indicating both PLGA micro/nanoparticles showed no innate cytotoxicity against the microorganisms. 1µg/ml CIP caused a 38.4% reduction of bacterial activity. The sustained release CIP from the PLGA micro/nanoparticles resulted in a bacterial activity reduction rate of 20.4% for PLGA microparticles and 25.8% for PLGA nanoparticles. According to these observations, the antibacterial activity of CIP encapsulated in PLGA nanoparticles was slightly better than that of the PLGA microparticles. As observed with the release studies, the microparticles had significant amounts of CIP remaining within the microspheres after several days, significantly longer than the time period of these standard antibacterial assays performed here and therefore may not reflect the actual in use performance.

To test the biological activity of CIP released from PLGA particles under OMF, drug released from microparticles and nanoparticles after 4 h OMF treatment was diluted to 1µg/ml then applied to *Pseudomonas aeruginosa* biofilms for 24 h. The bacterial activity reduction caused by CIP released from micro- and nanoparticles were 32.5% and 33.3% respectively. These reductions in bacteria were slightly lower compared with 1µg/ml free CIP control (38.4%). The slightly lower activity may be related to incomplete drug release, drug loss, or degradation during OMF. Elemental iron release from the MNP may also confound these observations and requires further investigation. Iron release may result higher antibacterial [45] but reports also suggest pseudomonas may also be supported by increased iron levels [46].

From these studies it was confirmed that CIP released from the particles before and after OMF triggered release was still active. The objective of this study was to investigate the release property of CIP encapsulated PLGA magnetic micro/nanoparticles, however in future studies formulations will be optimized and cytotoxicity will be tested.

## Conclusion

CIP encapsulated in PLGA magnetic micro/nanoparticles were successfully prepared using the W/O/W method. CIP encapsulated PLGA magnetic particles showed spherical shapes under a SEM, and narrow size distributions. In release study, there were three phases of drug release from PLGA magnetic micro/nanoparticles. The total drug release from nanoparticles (95%) was higher than that of microparticles (80.3%). In addition, CIP release from PLGA magnetic particles can be triggered in an external magnetic field and then continuously released for 2 weeks after triggered release. The total release amount under OMF was higher than passive release. A turn-on and -off switch experiment further illustrated the ability to have magnetically controlled drug release from PLGA particles. Activity against *P. aeruginosa* biofilms in vitro was confirmed. CIP released from PLGA particles under OMF retained antimicrobial activity. This study supports the potential application of PLGA magnetic particles as facilitators of drug delivery and drug release switches.
